# Editorial: Community series in mental illness, culture, and society: Dealing with the COVID-19 pandemic—Volume II

**DOI:** 10.3389/fpsyt.2022.1092845

**Published:** 2022-11-24

**Authors:** Samer El Hayek, Renato de Filippis, Mohammadreza Shalbafan

**Affiliations:** ^1^Medical Department, Erada Center for Treatment and Rehab, Dubai, United Arab Emirates; ^2^Psychiatry Unit, Department of Health Sciences, University Magna Graecia of Catanzaro, Catanzaro, Italy; ^3^Mental Health Research Center, Psychosocial Health Research Institute, Department of Psychiatry, School of Medicine, Iran University of Medical Sciences, Tehran, Iran

**Keywords:** coronavirus, COVID-19, frontline workers, health personnel, mental disorders, minorities, psychiatry, psychological impact

The COVID-19 pandemic has tremendously impacted our mental health (1, 2). Sociocultural factors, including norms, values, and religion, and individual factors have played an important role in shaping COVID-19-related psychiatric symptoms and disorders (3–6). These factors have also molded how mental health interventions are provided (7). As a continuation to Volume I of our Research Topic “*Mental Illness, Culture, and Society: Dealing with the COVID-19 Pandemic”* (8), in Volume II, we further investigate the intricate relationship between the pandemic and mental health. This volume particularly encompasses eight original articles, one brief report, and one systematic review, all highlighting this sophisticated association and how it can be moderated by sociocultural and individual variables.

Five studies directly looked at the differential impact of demographic, sociocultural, and other variables on COVID-19-related mental health symptoms. Using a cross-sectional design, Brooks et al. explored the relationship between demographic variables (i.e., ethnicity, sexual orientation, and disability status), mental health (i.e., distress, depression, anxiety, and somatic complaints), and vulnerability factors for COVID-19 (i.e., personal, community-related, and environmental) among 594 adults residing in the United States (US). Disparities were found for marginalized identities by gender, sexual orientation, and disability status. Younger individuals and those of lower economic status, across all identities, suffered more distress, depression, and anxiety (Brooks et al.). Pineros-Leano et al. looked at the impact of the pandemic on the mental health needs of Lantix families in the US. The authors conducted 21 semi-structured interviews with direct service providers working with Latinx communities. Five key themes were identified: worsening of mental health symptoms, economic stressors, preoccupation regarding transnational lives, secondary needs becoming more salient, and immigration status as a central driver of inequality. The authors raised the need to ensure access of Latinx immigrants to mental health services and the potential role of telehealth in achieving this (Pineros-Leano et al.). Shalaby et al. conducted a randomized controlled trial assessing the impact of Text4Hope services (*n* = 214; received once-daily supportive text messages for 6 weeks) vs. no intervention (*n* = 72; enrolled but did not receive messages) on mental health symptoms among males in Canada. Compared to controls, participants in the intervention group had significantly lower mean scores on the Perceived Stress Scale, Generalized Anxiety Disorder 7-item (GAD-7), Patient Health Questionnaire-9, and Composite Mental Health score. They also had a significantly lower prevalence of likely major depressive disorder (58.15 vs. 37.4%) and likely generalized anxiety disorder (50 vs. 30.8%) (Shalaby et al.). Alternatively, in their brief report, Razali et al. looked at factors associated with suicidal behaviors among 963 Malaysians during the first wave of the pandemic. Suicidal behavior was associated with gender, marital status, education, type of employment, residential area, number of people living together, number of children, and family dynamics. The authors concluded with recommendations for strategies to reduce and manage suicidality among vulnerable groups during the pandemic (Razali et al.). Lastly, using an online survey, Hu et al. compared stress levels during the first wave of the pandemic (*n* = 430) and one year later (*n* = 512) in China. They particularly looked at COVID-19-related stress, social support, and perceptions of the pandemic (perceived threat, perceived protection, and perceived controllability). Results indicated that Chinese people had lower COVID-19-related stress as the pandemic progressed. At both time points, more social support was associated with less stress. This was mediated by perceived protection and controllability of COVID-19 at both time points, and by perceived threat of COVID-19 during the first wave only (Hu et al.).

Four studies looked at the mental health of medical staff during the pandemic. Jing et al. investigated the incidence of post-traumatic stress disorder (PTSD), turnover intention, and psychological resilience of frontline medical staff (*n* = 443) in a public hospital in China. The total turnover intention and psychological resilience scores were 13.38 ± 4.08 and 87.16 ± 18.42, respectively. PTSD (incidence of 14.4%) was more prevalent among medical staff who were married, had children, and were worried about being infected. The PTSD group also had a higher level of education, higher turnover intention, and lower psychological resilience than the non-PTSD group. Lastly, higher scores on turnover intention and fear of being infected were significant risk factors for PTSD, whereas a higher education level and psychological resilience scores were significant protective factors (Jing et al.). In another original study, Kowalski et al. distributed a questionnaire to healthcare workers (*n* = 1,243) in Poland during four different waves of the pandemic. The Beck Depression Inventory (BDI-II), GAD-7, and Manchester Brief Assessment of Quality of Life (MANSA) scales were used. A gradual increase in moderate and severe anxiety and a decrease in fear due to the disease was observed as the pandemic progressed. No statistically significant differences were observed in comparing the mean values of the BDI-II, GAD-7, and MANSA scales across waves. Women, single people, and those with a psychiatric history were more likely to be affected (Kowalski et al.). Using a semi-structured interview, Arefin et al. analyzed the lived experiences of ten Bangladeshi frontline workers who were isolated after testing positive for COVID-19. Four primary themes and severe supporting themes emerged, including experience in a new working environment (e.g., maintaining social distance, misinformation, and fear of infection), diagnosis (e.g., experiences at the diagnosis center), recovery days (e.g., experiences in isolation and coping mechanisms), and post-COVID-19 (e.g., excitement, fear, and confusion, social stigma, and changes in philosophy) (Arefin et al.). Finally, in their meta-analysis of 23 studies (*n* = 27,325), Cheung et al. analyzed the psychological impact of the severe acute respiratory syndrome and COVID-19 epidemics in Asia on healthcare workers, as well as affected individuals and the general population. Findings showed high levels of mental health symptoms secondary to the outbreaks. In terms of the COVID-19 pandemic (12 studies), the overall prevalence rates of anxiety, depression, and stress were 34.8, 32.4, and 54.1%, respectively (Cheung et al.).

Shifting gears, in their cross-sectional study, Ghuloum et al. compared knowledge, attitudes, and practice related to COVID-19 infection between the public (*n* = 345), individuals attending outpatient psychiatry clinics (*n* = 165), and individuals admitted to psychiatry wards (*n* = 100). Results showed that, compared to the public, individuals with mental illness (inpatients and outpatients) had inadequate knowledge, more positive attitudes and confidence regarding COVID-19 outcomes, and fewer safe practices. Findings highlighted the need for a targeted approach among vulnerable individuals, particularly those with mental health problems (Ghuloum et al.).

In conclusion, Volume II of our Research Topic highlights, yet again, how the COVID-19 pandemic has worsened mental health symptoms throughout the globe; this has been mediated by a multitude of factors, including sociocultural, economic, and individual ones. Mental health experts should collaborate to provide timely, adequately tailored mental health treatment to those in need, particularly vulnerable groups.

## Author contributions

All authors listed have made a substantial, direct, and intellectual contribution to the work and approved it for publication.

## Conflict of interest

The authors declare that the research was conducted in the absence of any commercial or financial relationships that could be construed as a potential conflict of interest.

## Publisher's note

All claims expressed in this article are solely those of the authors and do not necessarily represent those of their affiliated organizations, or those of the publisher, the editors and the reviewers. Any product that may be evaluated in this article, or claim that may be made by its manufacturer, is not guaranteed or endorsed by the publisher.
